# T‐cell replete haploidentical stem cell transplantation with low dose anti‐thymocyte globulin for relapsed/refractory Ewing sarcoma family tumors

**DOI:** 10.1002/cnr2.1519

**Published:** 2021-07-22

**Authors:** Hideki Sano, Kazuhiro Mochizuki, Shogo Kobayashi, Yoshihiro Ohara, Nobuhisa Takahashi, Shingo Kudo, Kazuhiko Ikeda, Hitoshi Ohto, Atsushi Kikuta

**Affiliations:** ^1^ Department of Pediatric Oncology Fukushima Medical University Hospital Fukushima Japan; ^2^ Department of Blood Transfusion and Transplantation Immunology Fukushima Medical University School of Medicine Fukushima Japan

**Keywords:** Ewing sarcoma family tumor, graft versus tumor effect, refractory, relapse, T‐cell replete haploidentical stem cell transplantation

## Abstract

**Background:**

Despite intensive multimodal therapies, the prognosis of relapsed/ refractory Ewing sarcoma family tumors (RR‐ESFTs) is dismal. Some case reports using allogeneic stem cell transplantation (allo SCT) for RR‐ESFTs have been reported, however, the efficacy of allo SCT is yet to be established.

**Aim:**

The purpose of this study was to evaluate the response and toxicity of T‐cell replete haploidentical SCT (TCR‐haplo‐SCT) in RR‐ESFTs.

**Methods and results:**

In this study, we retrospectively analyzed six patients with RR‐ESFTs who received TCR‐haplo‐SCT. Four patients had relapsed and two patients had refractory Ewing sarcoma. Before the TCR‐haplo‐SCT, all patients received a reduced intensity‐conditioning regimen containing fludarabine, melphalan, and low‐dose rabbit anti‐thymocyte globulin (2.5 mg/kg), as well as graft‐versus‐host disease (GVHD) prophylaxis, which consisted of tacrolimus, methotrexate, and prednisolone. Primary neutrophil engraftment was achieved in all the patients. Four patients developed acute GVHD (aGVHD) (grade I, 1; grade II, 1; grade III, 2), and two patients developed chronic GVHD (cGVHD). Among the four that developed aGVHD, three survived for 14, 116, and 129 months without relapse, while one died due to a transplant‐related complication. In contrast, the two patients who did not develop aGVHD experienced relapse early after TCR‐haplo‐SCT.

**Conclusions:**

In this study, three of the six patients with RR‐ESFTs survived for more than one year without relapse, and the treatment toxicity was considered acceptable even for patients who underwent high‐intensity pretreatment. TCR‐haplo‐SCT could be a potential therapeutic option for patients with RR‐ESFTs.

## 1 INTRODUCTION

The 5 year survival rate of patients with relapsed/refractory Ewing sarcoma family tumors (RR‐ESFTs) is approximately 10%–20% despite intensive multimodal therapies.^1^, ^2^, ^3^, ^4^, ^5^ The age at relapse, time to relapse, pattern of relapse, and response to second‐line chemotherapy are considered as prognostic factors for survival. The reported 5 year overall survival rate for patients with relapse within 2 years of diagnosis was poor at 5%, while the 5 year overall survival rate for patients with relapse after 2 years was relatively good at 34.9%.^1^ Achieving second complete remission (CR) after relapse has been reported to be an important prognostic factor for survival.^6^ The reported probability of post‐relapse progression‐free survival at 2 years is around3% for patients who did not achieve a second CR.^6^ Allogeneic stem cell transplantation (allo SCT) has been presented as a potential curative approach based on the graft‐versus‐tumor (GVT) effect for intractable hematological malignancies. Even though there are case reports exploring and reporting the use of allo SCT in RR‐ESFTs,^7^, ^8^, ^9^ the efficacy of allo SCT for RR‐ESFTs is yet to be established. However, these reports suggested that allo SCT could improve the survival rates of RR‐ESFTs through cellular immunotherapy. Haploidentical SCT, which is associated with a stronger allogeneic immune response than conventional SCT, is rarely performed for solid tumors because of the high risk of transplantation‐related mortality (TRM) from graft‐versus‐host disease (GVHD) and the opportunistic infections.^10^ The curative role of haploidentical SCT and its impact on survival remains unclear.^11^ However, the authors confirmed that allo SCT was not associated with increased death from complications. In the current article, we report the outcomes of T‐cell replete haploidentical SCT (TCR‐haplo‐SCT) after reduced intensity conditioning for pediatric patients with RR‐ESFTs.

## 2 METHODS

Six patients with RR‐ESFTs who received TCR‐haplo‐SCT between 2010 and 2020 at Fukushima Medical University Hospital were retrospectively analyzed. Among the six patients, five were diagnosed with Ewing sarcoma, and one with primitive neuroectodermal tumor (PNET) (Table 1). The institutional review board approved the protocol, and written informed consent was obtained from the patients or their guardians, as well as their donor family members.

**TABLE 1:** Patient Characteristics

Patient No.	Age (years)	Sex	Initial Diagnosis	Initial Treatment	Disease status at completion of initial therapy	Time to relapse from initial diagnosis (months)	Relapse site	Post‐relapse treatment	HD‐chemo before TCR‐haplo SCT
1	23	M	localized ESFT	VDC‐IE, SR, RT	CR	26	lung, mediastinum	ICE, HD‐CY, RT	(−)
2	6	F	PNET	CY + DOX + VCR, SR, RT	microscopic residual disease	N/A	N/A	N/A	(−)
3	14	F	localized ESFT	DOX + IFO, VAC, RT	relapse	7	lung, BM, bone	VDC‐IE, RT	BU + MEL
4	23	F	localized ESFT	VDC‐IE, SR, RT	CR	41	lung, pleura	ICE	BU + MEL
5	15	F	localized ESFT	VDC‐IE, SR, RT	CR	26	skull base, lung	Topo + IFO	BU + MEL
6	15	M	metastatic ESFT	VDC‐IE, SR, RT	PR	N/A	N/A	N/A	BU + MEL

Abbreviations: BM, bone marrow; BU, busulfan; CR, complete remission; CY, cyclophosphamide; DOX, doxorubicin; ESFT, Ewing sarcoma family tumor; F, female; HD‐chemo, high dose chemotherapy; HD‐Cy, high dose cyclophosphamide; ICE, ifosphamide+carboplatin+etoposide; IE, ifosfamide+etoposide; IFO, ifosfamide; M, male; MEL, melphalan; N/A, not applicable; PNET, primitive neuroectodermal tumor; PR, partial remission; RT, radiation therapy; SR, surgical resection; TCR‐haplo SCT, T‐cell replete haploidentical stem cell transplantation; Topo, topotecan; VAC, vincristine+actinomycin‐D + cyclophosphamide; VCR, vincristine; VDC, vincristine + doxorubicin + cyclophosphamide.

Human leukocyte antigen (HLA) genotyping was conducted using PCR‐Luminex (Luminex Corporation, Austin, Texas), based on reverse sequence‐specific oligonucleotide (PCR‐rSSO) technology (Genosearch HLA, Medical & Biological Laboratories Co., Ltd., Nagoya, Japan) in Fukushima Medical University hospital. Peripheral blood stem cells (PBSCs) were collected from related donors using standard mobilization protocols. Granulocyte colony‐stimulation factor (G‐CSF) (400 μg/m^2^/day; Filgrastim, Kyowa Hakko Kirin Pharma Inc., Japan) was administered to the donors for five consecutive days to mobilize stem cells into the peripheral blood. PBSC harvesting was initiated on days 4 and 5 after G‐CSF administration. PBSC collection was performed using COBE Spectra or Spectra Optia (Terumo BCT, Tokyo, Japan).

The conditioning regimen for all patients consisted of fludarabine (30 mg/m^2^/day, days −9 to −5), melphalan (70 mg/m^2^/day, days −4 to −3), and low‐dose rabbit anti‐thymocyte globulin (thymoglobulin 1.25 mg/kg/day, days −2 to −1) (Table 2). GVHD prophylaxis was given with a combination of tacrolimus, short‐term methotrexate, and prednisolone.^12^ Methotrexate was administered intravenously, 10 mg/m^2^ on day +1, and 7 mg/m^2^ on days +3 and + 6 after transplantation. Prednisolone was started on day +0 at an initial dose of 1 mg/kg/day. If the patient showed no signs of acute GVHD (aGVHD), the initial dose was tapered every week from day +29 and was discontinued 2 months after transplantation. aGVHD and chronic GVHD (cGVHD) were graded using standard criteria.^13^, ^14^ Transplantation‐related toxicities were evaluated using the Common Terminology Criteria for Adverse Events (CTCAE version 4.0) outlined by the National Cancer Institute.^15^

**TABLE 2:** Graft and TCR‐haplo SCT related information and outcome

Patient No.	1	2	3	4	5	6
Disease status at TCR‐haplo SCT	SD (with large tumor burden)	microscopic residual disease	PR	PR	PR	PR
Conditioning	Flu + MEL + ATG	Flu + MEL + ATG	Flu + MEL + ATG	Flu + MEL + ATG	Flu + MEL + ATG	Flu + MEL + ATG
GVHD prophylaxis	PSL + TAC + MTX	PSL + TAC + MTX	PSL + TAC + MTX	PSL + TAC + MTX	PSL + TAC + MTX	PSL + TAC + MTX
Donor	mother	uncle	mother	father	mother	mother
HLA disparity (GVH direction)	2/8	3/8	3/8 with KIR ligand mismatch	4/8	3/8	3/8
Stem cell source	PBSC	PBSC	PBSC	PBSC	PBSC	PBSC
CD34 cell dose (×10^6^/kg)	4.9	12.4	3.6	5.6	4.9	5.9
CD3 cell dose (×10^8^/kg)	NE	NE	3.8	4	1.8	3
Neutrophil engraftment (day)	13	12	15	17	14	14
aGVHD Grade Stage (s,l,g)	none	I (1,0,0)	II (3,0,0)	III (1,0,2)	none	III (1,0,3)
cGVHD	NE	(−)	severe (GI tract score3)	NE	(−)	mild (skin score1)
Toxicity/Complication (<day100)	HHV6 encephalitis, radiation pneumonia	pancreatitis, CMV antigenemia	CMV antigenemia, BKV‐HC	EBV reactivation, Aspergilus	cystitis	CMV antigenemia, EBV reactivation, generalized convulsion
Toxicity/Complication (>day100)	NE	bladder dysfunction, ovarian dysfunction、	bone necrosis of femoral head, ovarian dysfunction, bladder dysfunction, anorexia	RSV pneumonia and encephalitis	none	depression, chronic gastritis, anorexia, bone necrosis of femur
Relapse or progression	(+)	(−)	(−)	(−)	(+)	(−)
TRM	(−)	(−)	(−)	(+)	(−)	(−)
RFS (mo)	4	129	116	4	6	14
OS (mo)	12	129	116	4	12	14
Disease status at last follow‐up	DOD	Alive with CR	Alive with CR	TRM	DOD	Alive with CR

Abbreviations: ATG, anti‐thymocyte globulin; BKV, BK virus; CMV, cytomegalovirus; CR, complete remission; DOD, dead of disease; EBV, Epstein–Barr virus; Flu, fludarabine; GI, gastrointestinal; GVHD, graft versus host disease; HC, hemorrhagic cystitis; HHV6, human herpes virus 6; HLA, human leukocyte antigen; KIR, killer cell immunoglobulin‐like receptor; MEL, melphalan; MTX, methotrexate; NE, not evaluable; OS, overall survival; PBSC, Peripheral Blood Stem Cell; PR, partial response; PSL, prednisolone; RFS, relapse free survival; SD, stable disease; TAC, tacrolimus; TCR‐haplo SCT, T cell replete haploidentical stem cell transplantation; TRM, transplantation related mortality.

## 3 RESULTS

### 3.1 Patient characteristics

Patient characteristics are shown in Table 1. The median age was 15 years (range, 6–23 years). The initial diagnoses included localized Ewing sarcoma (*n* = 4), metastatic Ewing sarcoma (*n* = 1), and PNET (*n* = 1). In the initial treatment, radiation therapy was administered in all cases, and all but one case (Patient 3) with primary pelvic tumor underwent surgical resection and chemotherapy. Disseminated relapse occurred during the initial treatment in one case (Patient 3). Two patients (Patients 2 and 6) did not achieve CR at the end of the initial treatment and were considered to have a poor prognosis; therefore, we decided to perform TCR‐haplo‐SCT. The other three patients achieved CR at the end of initial treatment, however, all of them relapsed with multiple lesions, and four relapsed patients (Patients 1, 3, 4, and 5) were treated with chemotherapy. Four patients (Patients 3, 4, 5, and 6) received high‐dose chemotherapy with busulfan and melphalan as consolidation therapy before TCR‐haplo‐SCT.

### 3.2 TCR‐haplo‐SCT related information

Detailed information regarding TCR‐haplo‐SCT is provided in Table 2. The disease status at the time of TCR‐haplo‐SCT was non‐CR in all patients. One patient had a large residual tumor mass in the lung and mediastinum that were unresponsive to chemotherapy. One patient had a microscopic residual tumor with positive post‐surgical margins. The remaining four patients responded to second‐line chemotherapy and experienced tumor shrinkage; however, metastatic tumors remained.

PBSCs were used as the stem cell source in all patients and were collected from family donors (four from mothers, one from a father, and one from an uncle). The patients received a median of 5.3 × 10^6^ CD34 positive hematopoietic cells/kg (range, 3.6 to 12.4 × 10^6^ cells/kg). We used unmanipulated PBSCs to avoid attenuation of the GVT effect. In the four evaluable patients, a median of 3.4 × 10^8^ CD3 positive cells/kg (range, 1.8–4.0 × 10^6^ cells/kg) were administered. All six patients achieved neutrophil engraftment. The median time to neutrophil engraftment was 14 days (range, 12‐17 days).

### 3.3 HLA disparities and GVHD

The HLA disparities to graft versus host directions were 2/8 in one patient (Patient 1), 3/8 in four patients (Patients 2, 3, 5, and 6), and 4/8 in one patient (Patient 4). In Patient 3, killer cell immunoglobulin‐like receptor (KIR) ligand mismatch was found in the HLA‐C between the donor and recipient. Acute GVHD was observed in four out of six patients (grade I: 1, II: 1, III: 2). Two patients with grade I and II aGVHD responded to temporal augmentation of corticosteroids. One patient (Patient 6) with grade III aGVHD was determined to be steroid‐resistant and was treated with infusions of mesenchymal stem cells (Temcell, JCR Pharma, Hyogo, Japan). Two patients (Patients 1 and 5) who did not have aGVHD died due to disease progression. Two of the four evaluable patients developed cGVHD, in which Patient 1 showed only mild skin symptoms. However, the other patient (Patient 3) suffered from severe cGVHD with severe gastrointestinal symptoms.

### 3.4 Complications within 100 days after the second TCR‐haplo‐SCT

Five patients had infectious complications within 100 days after the TCR‐haplo‐SCT, including cytomegalovirus antigenemia, Epstein–Barr virus reactivation, human herpes virus 6 encephalitis, and hemolytic cystitis due to BK virus. All were successfully treated with antiviral agents or by tapering their immunosuppression. Patient 1 developed pancreatitis (grade 2), and Patient 6 had a generalized convulsion (grade 2) of unknown origin.

### 3.5 GVT effect

**FIGURE 1:**
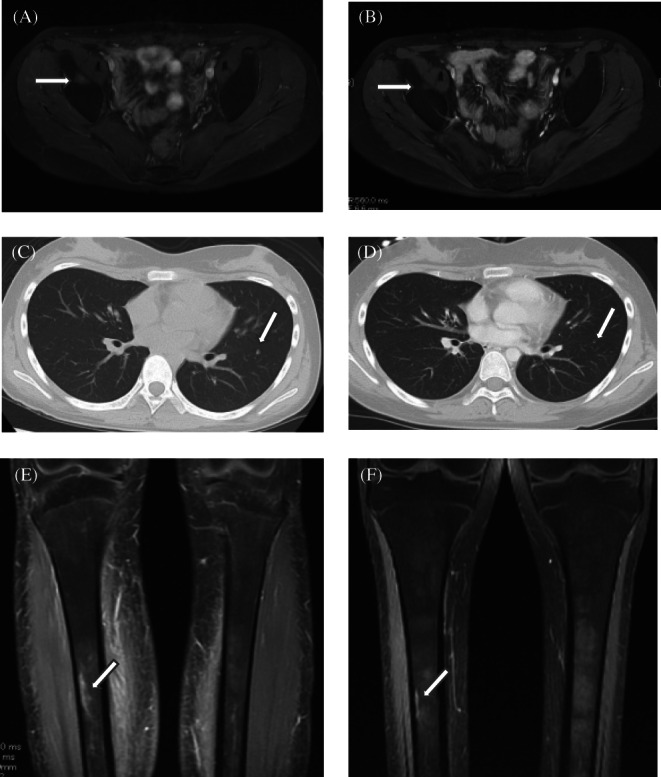
(A) Patient 3. Magnetic resonance imaging of the pelvic bone by short TI inversion recovery (STIR) prior to TCR‐haplo‐SCT. Arrow shows the bone metastasis of the right ilium. (B) Patient 3. Magnetic resonance imaging of the pelvic bone by STIR at 3 months after TCR‐haplo‐SCT. The size of the bone metastatic lesion in the right iliac bone was markedly reduced (arrow). (C) Patient 5. Computed tomography slice of lung prior to TCR‐haplo‐SCT. Arrow shows the lung metastasis of the left lung. (D) Patient 5. Computed tomography slice of lung at 3 months after TCR‐haplo‐SCT. The size of the left lung metastasis was reduced (arrow). (E) Patient 6. Magnetic resonance imaging of tibia by STIR prior to TCR‐haplo‐SCT. Arrow shows the bone metastasis of the right tibia. (F) Patient 6. Magnetic resonance imaging of tibia by STIR at 3 months after TCR‐haplo‐SCT. The lesion of the right tibia metastasis was reduced (arrow)

Results of imaging studies before and after the TCR‐haplo‐SCT in three patients with confirmed GVT effects are presented in Figure 1. The GVT effect was confirmed in iliac bone metastasis in Patient 3, lung metastasis in Patient 5, and tibia metastasis in Patient 6. Patient 2 was difficult to evaluate through imaging because of a residual microscopic lesion. Patient 4 died due to a transplantation‐related complication; therefore, we were unable to fully evaluate the case.

### 3.6 Transplantation‐related mortality, relapse, and outcome

One patient (Patient 4) had a severe respiratory syncytial virus infection after TCR‐haplo‐SCT and died on day 134 due to pneumonia and encephalitis. Two patients (Patients 1 and 5) died due to disease progression after TCR‐haplo‐SCT. The other three patients remained in remission for 14, 116, and 129 months after transplantation without relapse. There was one patient with bone marrow metastasis at disease onset (Patient 6) and one at relapse (Patient 3), but both are currently in remission. In particular, Patient 3, despite having disseminated relapse in the lung, bone, and bone marrow during initial treatment and also being presumed to have a poor prognosis, achieved long‐term survival. This patient's clinical course was reported by Yoshihara et al in 2016^9^ as a case report. Patients 3 and 6 experienced difficulty in ambulation due to osteonecrosis of the lower limbs caused by radiation therapy and long‐term steroid use.

### 3.7 Review of the literature on allogeneic HSCT for RR‐HSCT

While there have been no large clinical trials that have evaluated the efficacy of allogeneic HSCT for RR‐ESFTs, a few cases and small clinical studies have been reported.^7^, ^8^, ^11^, ^16^, ^17^, ^18^, ^19^ We have reviewed these articles and summarized them, with particular focus on the HSCT method and the associated conditioning regimen, in Table 3. A reduced intensity regimen with a busulfan, treosulfan or cyclophosphamide + fludarabine backbone appears to have been the preferred course of chemotherapy. In Europe, HLA haploidentical HSCTs were performed using CD34 positive selection or CD3 / CD19 depleted PBSCs. Our transplantation method was developed based on the reduced intensity regimen with Flu 150 mg/m2 + MEL 140 mg/m2 reported by Ueno et al in 2003^20^ for adult metastatic solid tumors. However, our protocol differed from those employed by other groups as we used low‐dose ATG (thymoglobulin 2.5 mg/kg) to control GVHD while preserving the GVT effect.

**TABLE 3:** Literature review on patients with RR‐ESFT who underwent allogeneic hematopoietic stem cell transplantation

N	Disease features	Stem cell source	Conditioning	GVHD prophylaxis	CD34 cell dose (×10^6^/kg)	Outcome	References
1	relapse	CD34 + PBSC from HLA mismatched relative	BU + TT + FLU + CY + anti‐CD3 antibody	no	19.1	42 months + (tumor regression after severe GVHD induced by low dose IL‐2 therapy)	^7^
1	relapse	PBSC from HLA matched relative	BU 12.8 mg/kg + MEL 180 mg/m^2^ + thymoglobulin 9 mg/kg	CyA + sMTX	3.5	9 months +	^8^
1	relapse	CD3 / CD19 depleted PBSC from HLA mismatched relative with KIR ligand mismatch	BU 8 mg/kg + TT 10 mg/kg + FLU 150 mg/m^2^	mPSL + sMTX + CyA	5.8	DOD at 9 months	^18^
1*	relapse	CD3 / CD19 depleted PBSC from HLA mismatched relative	TREO + TT + FLU + anti‐CD3 antibody	MMF	9.5	50 months +	^19^
7	metastatic	6 from HLA mismatched relative 1 from matched sibling	FLU 150 mg/m^2^ + TT 10 mg/kg + MEL 70 mg/m^2^	unknown	unknown	median DFS 28 months (range 11–73)	^11^
11	relapse 4 refractory 2 metastatic 5	PBSC from HLA matched relative	CY 3600 mg/m^2^ + FLU 120 mg/m^2^ + MEL 100 mg/m2	CyA or Tac + Sirolimus	unknown	median OS 15.8 months (range 4–77)	^16^
4	relapse 2 CR1 2	PBSC from HLA mismatched relative	CY 29 mg/kg + FLU 150 mg/m^2^ + MEL 100 mg/m^2^ + TBI 200 cGy	Post‐CY 50 mg/kg × 2 + MMF + Sirolimus	unknown	Relapsed; died at 21.4 and 14.6 months CR1; alive at 15.7 and 9.2 months	^26^

Abbreviations: BU, busulfan; CY, cyclophosphamide; CyA, cyclosporine; DFS, disease free survival; DOD, dead of disease; ESFT, Ewing sarcoma family tumor; FLU, fludarabine; GVHD, graft versus host disease; IL‐2, interleukin‐2; MEL, melphalan; MMF, mycophenolate mofetil; OS, overall survival; PBSC, peripheral blood stem cell; sMTX, short course methotrexate; Tac, tacrolimus; TREO, treosulfan; TT, thiotepa.

^*^ Two cases were reported, but one case may have been already reported in reference 7.

## 4 DISCUSSION

There has been no significant improvement in outcomes for RR‐ESFTs in the past two decades, and new breakthroughs are needed to improve survival. In recent years, immune checkpoint inhibitors utilizing autologous cytotoxic T‐cells have shown effectiveness against treatment‐relapsed/resistant solid tumors such as melanoma, renal cell carcinoma, lung cancer, and breast cancer. However, their efficacy against sarcomas has not been confirmed.^21^ A clinical study using insulin‐like growth factor 1 receptor antibody demonstrated little efficacy as a single‐agent treatment.^22^ In contrast, there have been several case reports showing the effectiveness of allo SCT against RR‐ESFT.^7^, ^8^, ^9^ Therefore, we hypothesized that the use of TCR‐haplo‐SCT, which as a strong graft‐versus‐leukemia effect on refractory leukemia,^23^ could similarly serve as a novel immunotherapy for RR‐ESFT through GVT effects mediated by alloreactive T‐cells. Given that past studies have shown increased TRM in heavily pre‐treated solid tumor patients undergoing myeloablative allo SCT,^24^ we chose non‐myeloablative conditioning to minimize TRM and optimize efficacy through GVT effect.

Burghuis et al^25^ reported that complete or partial lack of HLA class I expression was found in 79% of Ewing sarcoma tumors and that HLA class I expression decreases with disease progression. Simultaneously, they showed that HLA class I expression is restored by IFN‐γ.^25^ This may provide a theoretical basis for the relapse of two patients without GVHD among the six patients that we reported in this study. Interestingly, Schober et al^17^ performed donor lymphocyte infusion (DLI) after allo SCT for advanced ESFT or rhabdomyosarcoma and reported that HLA‐mismatched DLI showed longer post‐relapse survival than HLA‐matched DLI (23 vs 3 months). They concluded that pre‐emptive DLI should be administered in the absence of GVHD after allogeneic SCT for high‐risk pediatric ESFT or rhabdomyosarcoma.

Acute GVHD has also been reported in several case reports of successful allogeneic transplantation. Koscielniak et al^7^ reported a case of a female patient with relapsed ESFT, who underwent haploidentical PBSC transplantation from her mother with HLA 2 locus mismatch. After transplantation, low‐dose interleukin‐2 was administered to induce aGVHD, which thereby induced the GVT effect and achieved long‐term survival by developing grade IV aGVHD. The case reported by Lucas et al^8^ also showed skin aGVHD and limited cGVHD.

The clinical impact of KIR ligand mismatch on the outcome of hematopoietic SCTs in ESFT remains unclear. Although KIR ligand mismatch and long‐term survival were observed in Patient 3 of our study, the GVT effect was limited in ESFT after SCT with KIR ligand mismatch, as reported by Pérez‐Martínez et al^18^ We believe that the allogeneic immune response by cytotoxic T‐cells is the main cause of the antitumor effect in TCR‐haplo‐SCT, since the GVT effect coincides with the expression of aGVHD. In fact, in a clinical trial of reduced‐intensity haploidentical bone marrow transplantation for pediatric solid tumors using post‐transplant cyclophosphamide, which strongly suppresses the allogeneic immune response, 1‐year progression‐free survival was relatively poor at 16%.^26^ However, it should be noted that our HSCT method may be associated with a high risk of severe aGVHD and resulting complications. In addition, cases of acute leukemia relapse after haplo‐HSCT due to loss of mismatched HLA haplotype have been reported,^27^ and there is a possibility that a similar pattern of recurrence may occur in solid tumors. In light of this problem, it is worth considering the results of studies conducted from a different perspective on HLA‐haplo‐HSCT as an immune cell therapy for ESFT. Schlegel et al^19^ analyzed NK cell‐mediated antitumor activity in two ESFT patients who underwent haploidentical HSCT with ex vivo T‐cell depletion. They found that alloreactive NK cells stimulated with cytokines (such as IL2 / IL15) and an anti‐GD2 CH14.18 monoclonal antibody to induce antigen‐dependent cellular cytotoxicity significantly increased the lysis of ESFT cell lines. These authors are conducting a prospective clinical trial based on this strategy for recurrent / refractory neuroblastoma,^28^ but it is unclear how effective this approach might be for ESFT.

Thiel et al^29^ compared survival rates after hematopoietic allo SCT of HLA‐mismatched versus HLA‐matched advanced Ewing sarcoma patients and reported no significant difference. However, they performed ex vivo T‐cell depletion in haploidentical transplantation, which is different from our haploidentical SCT method. In fact, there was no significant difference in the frequency of aGVHD between HLA‐mismatch and HLA‐matched groups in this study. They also found that disease status prior to hematopoietic allo SCT was the strongest prognostic factor with a significantly lower hazard ratio (0.4) for patients who achieved CR at transplantation. In our study, no GVT effect was observed in Patient 1, who had a large tumor burden, suggesting that pre‐transplant disease status might be the greatest effect on transplantation outcome. Therefore, for successful TCR‐haplo‐SCTs, it is essential to first reduce the tumor volume through multidisciplinary treatments after relapse. While our method did not exhibit a therapeutic effect on large residual tumors, it may be possible to increase the anti‐tumor effects by combining it with drugs that inhibit the immune escape mechanism of the tumor, such as immune checkpoint inhibitors.

Although the sample size of this study was small, our data suggests that TCR‐haplo‐SCTs for RR‐ESFTs are feasible and demonstrated three evaluable patients with GVT effect. We believe that TCR‐haplo‐SCT could be a potential therapeutic option for patients with RR‐ESFTs. Further clinical studies are required to determine the efficacy of this novel approach.

## CONFLICT OF INTERESTS

The authors declare that they have no conflicts of interest related to this study.

## ETHICAL STATEMENT

All procedures performed in studies involving human participants were in accordance with the ethical standards of the institutional research committee and with the 1964 Helsinki Declaration and its later amendments or comparable ethical standards. This retrospective study was approved by the institutional review board.

## AUTHOR CONTRIBUTIONS

All authors had full access to the data in the study and take responsibility for the integrity of the data and the accuracy of the data analysis. *Conceptualization*, H.S., A.K.; *Methodology*, K.I., H.O., H.S.; *Investigation*, K.M., S.K., Y.O., N.T., S.K.; *Resources*, K.I., H.O., H.S.; *Writing‐Original Draft*, H.S. *Writing‐Review & Editing*, H.S., K.M., S.K., Y.O., N.T., S.K., K.I., H.O., A.K.; *Project Administration*, H.S., A.K.

## Data Availability

The data used in this study are not publicly available because of privacy and ethical restrictions.
